# Ilizarov external fixation for a periprosthetic tibial fracture in severe osteoporosis: a case report

**DOI:** 10.1186/s12891-020-3176-x

**Published:** 2020-03-04

**Authors:** Koji Nozaka, Naohisa Miyakoshi, Takeshi Sato, Yoichi Shimada

**Affiliations:** Department of Orthopedic Surgery, Akita University Graduate School of Medicine, 1-1-1 Hondo, Akita, 010-8543 Japan

**Keywords:** Periprosthetic tibial fracture, Total knee arthroplasty, Ilizarov external fixator

## Abstract

**Background:**

The incidence of periprosthetic fractures after total knee arthroplasty (TKA) is rising due to the increasing number of TKAs performed annually and the growing elderly population. A periprosthetic fracture of the proximal tibia following TKA is a rare injury that may be a challenging clinical scenario.

**Case presentation:**

The case of an 84-year-old woman who sustained a periprosthetic tibial fracture 10 years after a TKA is presented. This patient had multiple risk factors. The fracture was not deemed amenable to conventional treatment because the bone fragment was too small. This patient underwent fixation of her tibial fracture above the TKA using a five-ring Ilizarov external fixator. This allowed immediate full weight-bearing. The fixator was removed at 12 weeks, at which time the fracture was solidly healed. At the most recent follow-up, 2 years from injury, she was fully weight-bearing without walking aids and had a knee range of motion (ROM) of 0–110°.

**Conclusion:**

To the best of our knowledge, this is the first report in which Ilizarov external fixation has been used for a periprosthetic tibial fracture after TKA.

## Background

The incidence of periprosthetic fractures after total knee arthroplasty (TKA) is rising due to the increasing number of TKAs performed annually and the growing elderly population. Periprosthetic fractures after TKA are an increasing problem and challenging to treat. Periprosthetic fractures of the tibia, or fractures below TKAs, are less common than periprosthetic fractures of the distal femur. However, the literature on the outcomes of periprosthetic tibial fractures treated with modern techniques is limited.

## Case report

**Fig. 1:**
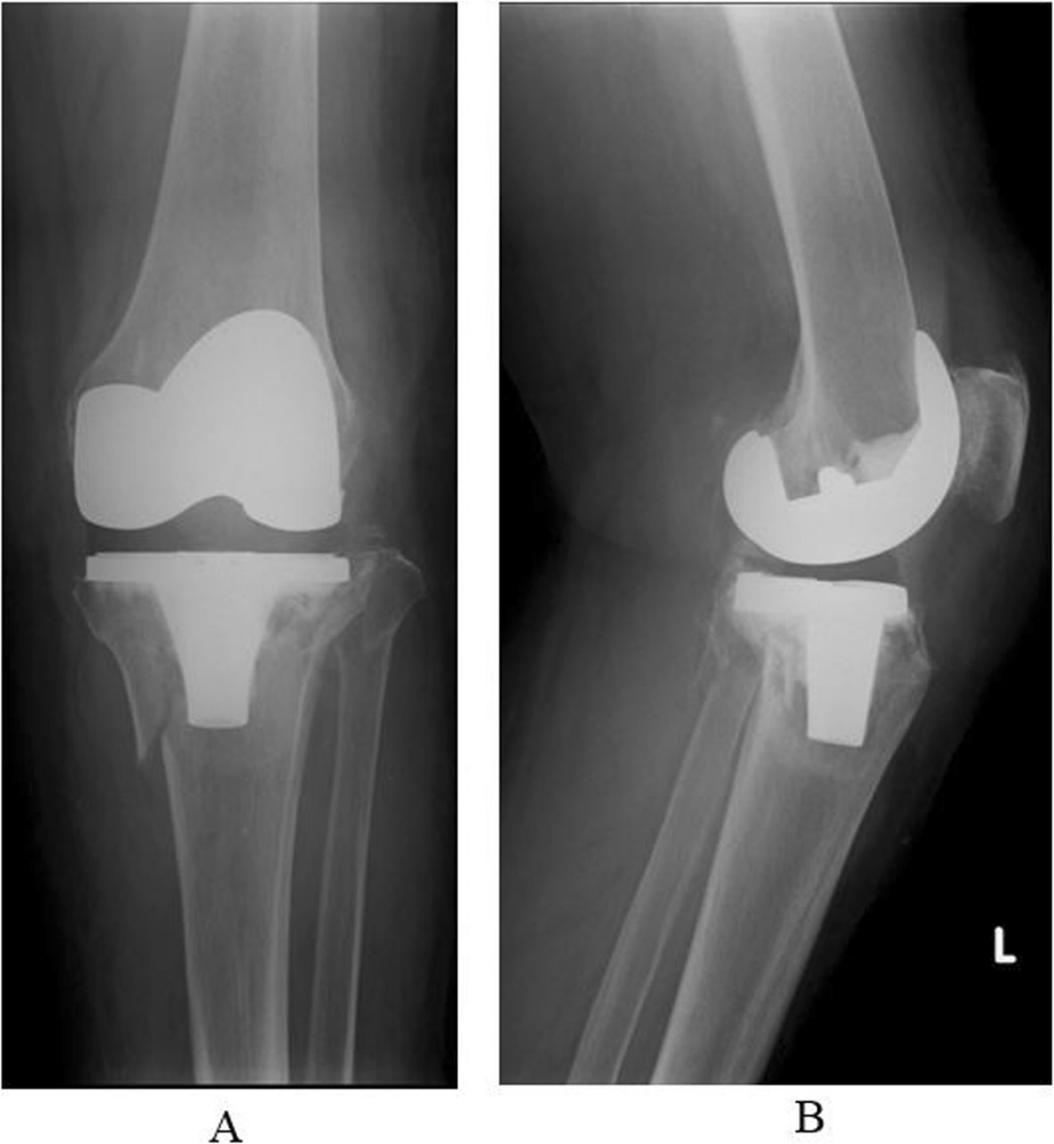
**a** Preoperative antero-posterior X-ray. **b** Preoperative lateral angle X-ray

The case of an 84-year-old woman who sustained a periprosthetic tibial fracture as a result of a fall from standing height (Felix classification type IIA [1]) 10 years after a left TKA is presented (Fig. 1). A posterior-stabilized total knee prosthesis (NexGen LPS-Flex system) was used. The patient was unable to walk due to severe knee pain after the injury. She had severe rheumatoid arthritis (Steinbrocker class III), hypertension, diabetes mellitus, and glucocorticoid-induced osteoporosis. Her medications included methotrexate 8 mg/week, prednisolone 10 mg/day, and intravenous alendronate once monthly. A baseline dual-energy X-ray absorptiometry scan showed that her femoral neck bone mineral density was 0.31 g/cm^2^. The patient was also at high risk for general anesthesia due to severe heart failure and renal failure. She was neurovascularly intact distally. Thus, the decision was made to apply a circular thin-wire external fixator with only an epidural block.

**Fig. 2:**
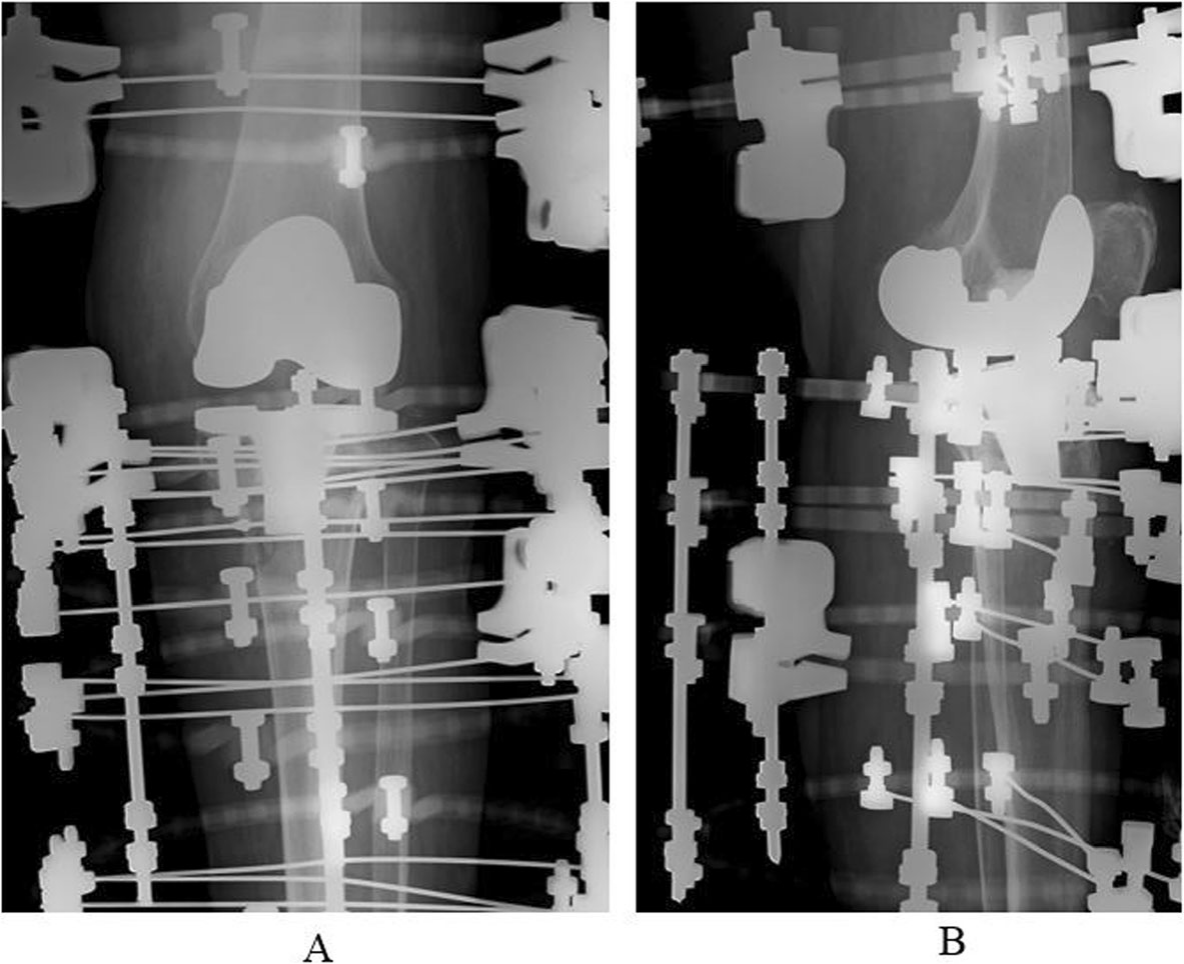
**a** Postoperative antero-posterior X-ray. **b** Postoperative lateral angle X-ray

**Fig. 3:**
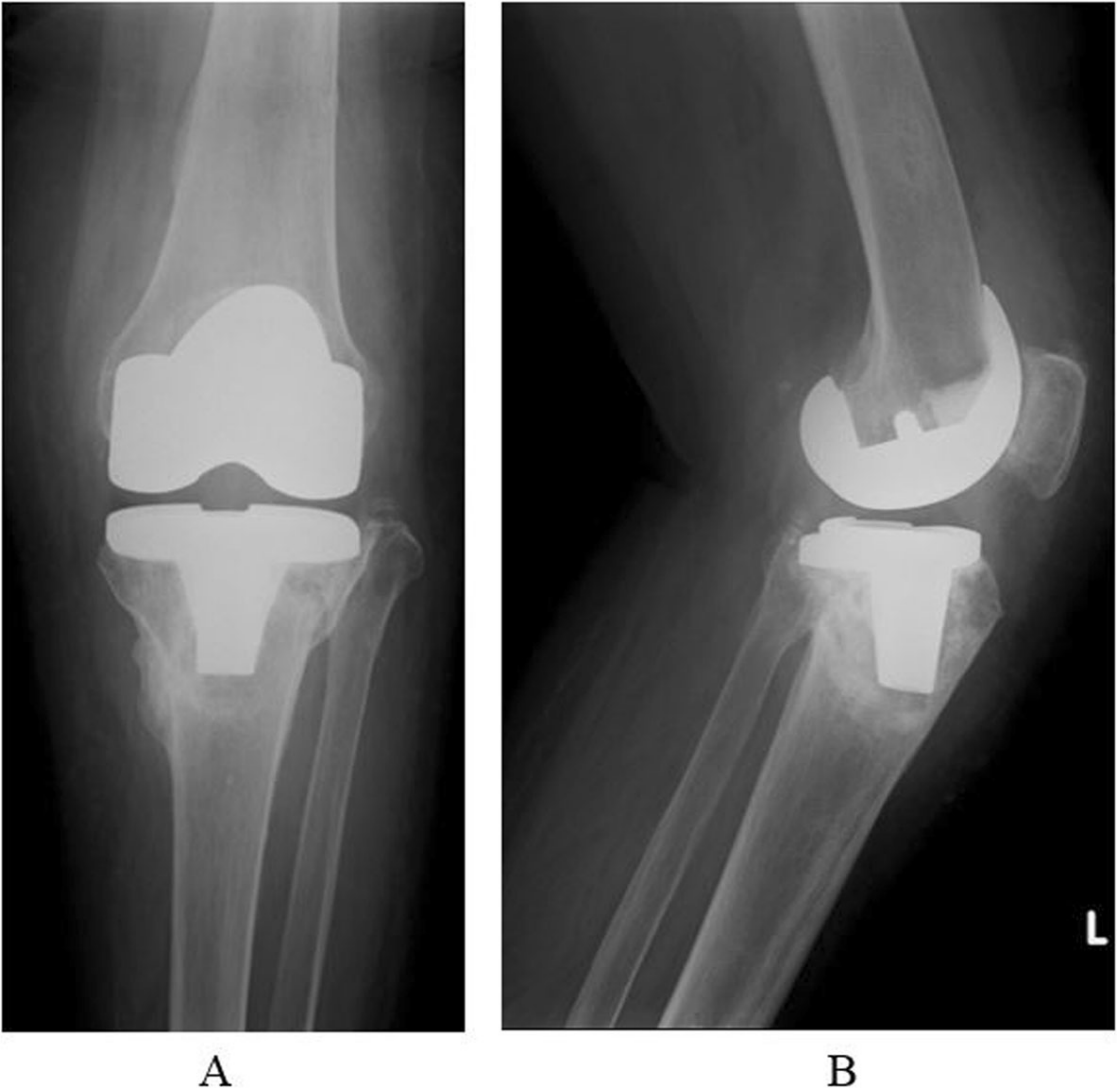
**a** After ring removal, antero-posterior X-ray. **b** After ring removal, lateral angle X-ray

A five-ring Ilizarov external fixator was applied, using thin wires (1.8 mm) under fluoroscopic guidance. The frame was placed to span the knee joint. Postoperative radiographs showed satisfactory reduction of the fracture fragments (Fig. 2). The total operating time was 90 min. The patient began full weight-bearing immediately, and knee range of motion (ROM) exercises were started at postoperative 2 weeks after femoral ring removal. Radiographs at 8 weeks showed good callus formation. The fixator was removed at 12 weeks. Though the bone defect vacancy had sunk after reduction, and the posterior tilt was increased, there were no particular clinical symptoms due to the increased posterior tilt (Fig. 3). This patient was allowed to walk with full weight-bearing immediately after surgery. A knee joint-spanning external fixator was used, the femoral ring was removed 2 weeks after surgery, and ROM exercises were started. At follow-up, 14 months after injury, she was mobilizing independently, with a knee range of movement of 0–110°. During the 12 weeks in the fixator, there was only one superficial pin-tract infection, which was treated with empirical oral antibiotics and daily pin-tract dressings.

### Surgical technique

**Fig. 4:**
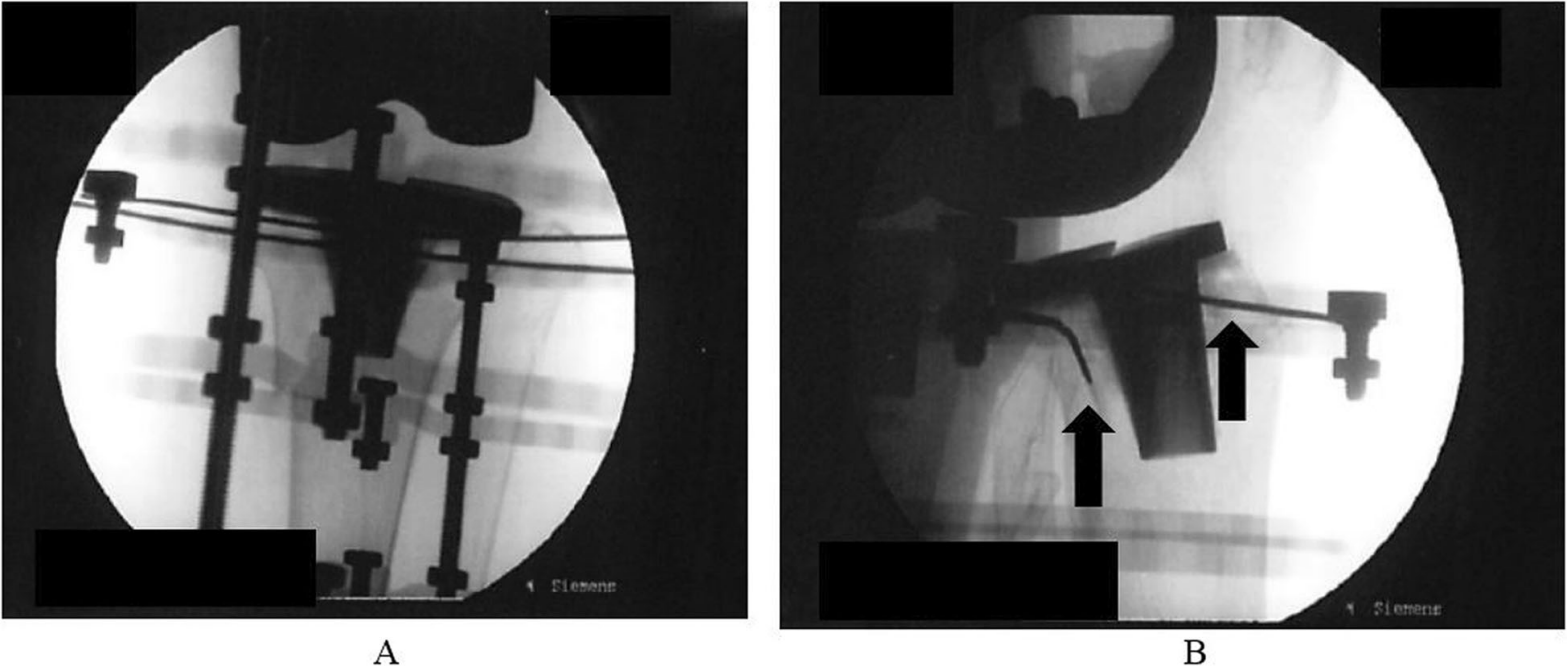
**a**, **b** The surgeon uses the lateral fluoroscopic view to identify a safe starting point and inserts the wire from the front and back of the tibial TKA component (arrow) without touching the stem

There was fracture site instability and a medial spike. Therefore, the skin on the medial side of the fracture site was in poor condition. Reduction by manual manipulation alone was impossible. First, two straight wires were inserted into the proximal tibia and attached to the proximal 5/8 ring. The surgeon used the lateral fluoroscopic view to identify a safe starting point and inserted the wire from the front and back of the tibial TKA component without touching the stem (Fig. 4). Two straight wires were then inserted into the distal tibia and attached to the full ring. The middle two full rings were left free near the distal ring so that they did not interfere when checking reduction of the fracture area. An assistant held the proximal ring, and the surgeon moved the distal tibia ring anchored to the proximal tibia in distraction, flexion, extension, valgus, and varus. This maneuver was gently and carefully repeated over time. By relieving the “jamming” of the fracture area by longitudinal traction with a large force with the tibial ring, most of the dislocation could be reduced by closed manipulation with accurate alignment. By using ligamentotaxis, reduction was possible from the outside of the body.

After reduction, the two full rings, which were left free between the proximal ring and the most distal ring, were moved near the fracture site and fixed with straight wires. In addition, three straight wires were added to the proximal ring in the tibia for increased fixation.

Finally, the parts of the rods for ligament traction that protruded distally were cut so that they did not interfere with rehabilitation.

## Discussion and conclusions

Periprosthetic fractures of the tibia, or fractures below TKAs, are less common than periprosthetic fractures of the distal femur, with a prevalence of between 0.4 and 1.7% [1–3]. Many authors have reported several fixation options to treat these difficult fractures [4–7]. However, no consensus exists regarding the optimal fixation for periprosthetic tibial fractures.

This case did not require a skin incision for reduction, and the osteosynthesis could be completed by closed reduction with an extracorporeal operation using the ligament traction method with an Ilizarov external fixator. The frame was constructed to span the knee joint, with the joint held in extension. After 2 weeks, the patient started knee ROM exercises after femoral frame removal.

An Ilizarov external fixator is an adequate treatment option that a surgeon should have in mind for the management of very elderly and debilitated patients with periprosthetic tibial fractures.

There have been some reports of Ilizarov external fixation for the treatment of periprosthetic fractures of the distal femur [7, 8]. Robin et al. reported that an Ilizarov external fixator is indicated in an elderly patient with periprosthetic fractures of the distal femur with extremely osteopenic bone [8].

Some studies have reported that a major advantage of Ilizarov external fixation is the ability to achieve rigid fixation for osteoporotic bones, which can be obtained through the insertion of multiple thin, straight wires [7–9]. Beris et al. reported that Ilizarov external fixation is a feasible and effective treatment option, because it provides stable fixation, prompt postoperative mobilization, and has no major complications, especially in elderly patients treated for periprosthetic fractures [7]. Furthermore, gentle closed reduction and fixation are beneficial for effective bone union in terms of biological characteristics and vascularization of the fracture area [10]. Since long-term non-weight-bearing leads to reduced walking ability in older patients, walking with an Ilizarov external fixator with strong fixation immediately after surgery may greatly benefit them, and mechanical stimulation by weight-bearing may have additional effects [11].

There is a risk of pin-tract infections with this approach. When treating periprosthetic fractures around the knee with an Ilizarov external fixator, meticulous pin care and immediate treatment with antibiotics are necessary at any sign of infection [8]. In addition, walking with an Ilizarov external fixator is difficult. However, this method is very promising because it is minimally invasive with low intraoperative blood loss and minimal patient discomfort.

Schreiner et al. reported that periprosthetic tibial fractures predominantly affect elderly patients with reduced bone quality and have a high complication rate [12]. Osteoporosis makes the use of internal fixation devices for periprosthetic tibial fractures more challenging in elderly patients [9, 12].

Ilizarov external fixation provides stable fixation, prompt postoperative mobilization, and has no major complications. It provides the postoperative capability for malalignment correction, and in the hands of an expert, Ilizarov external fixation is not time-consuming [8, 13].

In the present case, the fracture occurred in a patient with severe rheumatoid arthritis, osteoporosis, and heart failure. Kim et al. reported that osteoporosis, such as that which occurs with old age, rheumatoid arthritis, and use of corticosteroids, is a risk factor for periprosthetic tibial fractures [4].

Treatment options for periprosthetic tibial fractures include using a knee immobilizer, traction, casting, open reduction and internal fixation with a plate-and-screw construct, revision arthroplasty, and amputation [14–18]. In the present case, the decision to place an Ilizarov external thin-wire fixator as minimally invasive surgery was made due to the patient’s severe heart failure and renal failure. With periprosthetic tibial fractures in cases of severe osteoporosis, there is the option of revision arthroplasty with a long stem, but this was not deemed suitable in the present case due to the high risk of general anesthesia. Open reduction and internal fixation were considered, but poor bone quality and the relatively small distal fragment, which would not allow adequate screw fixation, resulted in the Ilizarov external fixator being used. The decision was made to place the device to allow immediate full weight-bearing.

## Data Availability

The datasets used and/or analyzed during the current study are available from the corresponding author on reasonable request.

## Abbreviations

ROM: Range of motion
TKA: Total knee arthroplasty
